# Sirtuin 3 deficiency exacerbates age‐related periodontal disease

**DOI:** 10.1111/jre.12930

**Published:** 2021-09-30

**Authors:** Junsheng Chen, Yarong Zhang, Jing Gao, Tao Li, Xueqi Gan, Haiyang Yu

**Affiliations:** ^1^ State Key Laboratory of Oral Disease National Clinical Research Center for Oral Diseases West China Hospital of Stomatology Sichuan University Chengdu China; ^2^ Faculty of Medicine and Dentistry Department of Cell Biology University of Alberta Edmonton Alberta Canada; ^3^ West China–Washington Mitochondria and Metabolism Center and Department of Anesthesiology West China Hospital Sichuan University Chengdu China

**Keywords:** mitochondria, oxidative stress, periodontal disease, SIRT3

## Abstract

**Background:**

Sirtuin 3 (SIRT3), a mitochondrial NAD^+^‐dependent deacetylase, has received much attention for its effect on metabolism and aging. However, the role of SIRT3 in periodontal disease remains unknown.

**Objective:**

This study aimed to investigate the functional role of SIRT3 in age‐related periodontal disease and underlying mechanisms.

**Methods:**

Sixteen mice were randomly assigned into four groups: the young wild type (WT), the aged WT, the young SIRT3‐knockout (KO), and the aged SIRT3‐KO. SIRT3 and cyclophilin D (CypD) expression and protein lysine acetylation levels in alveolar bones were detected by western blot. The bone architecture and the distance of CEJ‐ABC were assessed using micro‐CT and HE staining. The osteoclast number was observed through tartrate‐resistant acid phosphatase (TRAP) staining. Mitochondrial morphology in SIRT3 knockdown MC3T3‐E1 osteoblastic cells was analyzed by Immunofluorescence staining. In gingival tissues, the NAD^+^/NADH ratio was measured, and oxidative stress was detected by MitoSOX staining, HO‐1 staining, and MnSOD expression. Mitochondrial biogenesis was measured by PGC‐1α expression and oxygen consumption rate (OCR).

**Results:**

In parallel with the imbalanced NAD^+^/NADH ratio, the SIRT3 expression was significantly decreased in the alveolar bones of the aged mice, accompanied by a global elevation of protein acetylation levels. The aged SIRT3‐KO group showed the highest rate of bone resorption and the largest number of TRAP‐positive osteoclasts among the four groups. Moreover, the reactive oxygen species level was up‐regulated in the young and the aged SIRT3‐KO groups. SIRT3 deficiency promoted mitochondrial fission and increased the CypD expression. Furthermore, the lack of SIRT3 reduced the PGC‐1α expression in gingival tissues and exhibited a significant reduction in maximal OCR.

**Conclusion:**

Reduced SIRT3 abundance contributes to aged‐related periodontal disease via the exacerbation of oxidative stress and mitochondrial dysfunction.

## 1 INTRODUCTION

Aging, a common phenomenon to all multicellular organisms, is described as an endogenous and progressive decay during the whole life span.^1^ Some chronic diseases, such as diabetes, cancer, cardiovascular disease, and neurodegenerative disease, are associated with chronological aging. Periodontal disease, also related to age, is an infectious disease resulting in inflammation within the tooth‐supporting tissues, progressively diminished tooth attachment and alveolar bone loss.^2^, ^3^

The “Mitochondrial Theory of Aging” posits that a decline in mitochondrial function over time is an underlying cause of aging.^4^, ^5^ Sirtuins (SIRT1–7) are a family of nicotinamide adenine dinucleotide (NAD^+^)‐dependent protein deacetylases^6^ that regulate multiple cellular processes.^7^, ^8^ SIRT3 is a major mitochondria‐localized deacetylase and controls mitochondrial protein acetylation,^9^, ^10^ participating in the progress of human aging.^11^ It initiates robust deacetylase activity toward a series of metabolic targets, including subunits of the electron transport chain (ETC), as well as enzymes involved in redox balance, tricarboxylic acid cycle, and ketone body production.^12^, ^13^, ^14^, ^15^

Reactive oxygen species (ROS) is a natural by‐product of cellular respiration,^16^ that increases dramatically with age.^17^ Mitochondria are both the major source and principal attack target of ROS.^18^ A previous study reported that SIRT3 can reduce ROS levels by modulating key antioxidant enzymes directly, acting as a shield against oxidative damage.^11^ At higher ROS levels, longer mitochondrial permeability transition pore (mPTP) openings may release a ROS burst leading to mitochondria destruction.^19^ Cyclophilin D (CypD), an undisputed regulator of the mPTP, is located in the mitochondrial matrix, which can be translocated to the inner mitochondrial membrane.^20^ The evidence indicates that CypD can be deacetylated by SIRT3, resulting in an inhibited mPTP opening,^21^, ^22^, ^23^ but whether SIRT3 regulates CypD involved in the pathogenesis of periodontal disease has not been evaluated.

Although the crucial role of SIRT3 has been confirmed, limited in vivo studies have explored its specific effects in periodontal tissues. The current study was designed to investigate the functional role of SIRT3 in the pathogenesis of age‐related periodontal disease and to explore underlying mechanisms.

## 2 MATERIAL AND METHODS

### 2.1 Animals

All animal experiments were performed with the approval of the Institutional Animal Care and Use Committee of the West China Hospital, Sichuan University, China. The SIRT3 germ‐line knockout (KO) mice with 129SV background were purchased from Jackson Laboratory. Mice at 3 months (young) or 16 months of age (aged) were used and kept on a 12:12‐h light–dark cycle at 22°C with water and food ad libitum.

### 2.2 Cell culture and treatment

Murine MC3T3‐E1 osteoblastic cells were cultured in α‐MEM medium (Gibco) containing 10% FBS and 1% penicillin–streptomycin in 6‐well plates, under 5% CO2 and 37°C. Cells were divided into the control (shCtrl) group and the knockdown (shSIRT3) group by incubation with control or SIRT3^−/−^ shRNA (GenePharma) for 48 h, respectively.

### 2.3 Western blot analysis

The frozen maxillary samples were washed by cold PBS three times, and then homogenized in lysis buffer (140 mM NaCl, 1 mM ethylenediaminetetra‐acetic acid (EDTA), 10% glycerol, 1% NP40, 20 mM Tris‐HCl, pH 7.5, containing protease inhibitor, 1 mM PMSF). After centrifugation, the supernatant was separated and applied to western blot analysis. A total of 20 μg of protein was loaded to 10% SDS‐PAGE and transferred to PVDF membranes (Bio‐rad). After being blocked in 5% non‐fat milk for 1 h, the membranes were incubated at 4°C overnight with different primary antibodies including anti‐SIRT3 (1:1000, #510962, ZENBIO), anti‐cyclophilin 40 (1:1000, #ab181983, Abcam), anti‐acetylated‐lysine antibody (1:1000, #9441S, CST) and GAPDH (1:10000, #ab8245, Abcam). After that, the membranes were incubated with secondary anti‐rabbit IgG antibody (1:10000, #ab6721, Abcam) or anti‐mouse IgG antibody (1:5000, #ab6789, Abcam). Immuno‐reactive protein bands were visualized using a chemiluminescence machine (Bio‐Rad,). Band relative optical density was detected by NIH Image J software and normalized with GAPDH levels.

### 2.4 Micro‐CT analysis and alveolar bone loss measurement

The harvested right mandibles were fixed in 10% formaldehyde for 72 h, and then scanned by μCT 50 (Scanco Medical) under medium resolution with a voxel size of 10 μm and an energy setting of 90 kV, 114 mA, and an integration time of 500 ms. Raw images were reconstructed and analyzed with SCANCO Medical Evaluation and Visualizer software. The region of interest (ROI) was defined as the buccal–lingual crossline of the first mandibular molar in the furcation zone (between the mesial and distal roots). Ten slices before and after the identified furcation slice were added to generate a ROI, and the bone volume per total volume (BV/TV, %) was calculated within it. After scanning, the degree of alveolar bone resorption was evaluated by the three‐dimensional image analysis software, in accordance with the previous study.^24^ The distance from the cemento‐enamel junction (CEJ) to the alveolar bone crest (ABC) of the first molar was measured, and the average of six sites (mesio‐buccal, buccal, disto‐buccal, disto‐lingual, lingual, and mesio‐lingual) was used as a measurement of bone loss.

The mandibles were then air dried. Each jaw was oriented and the distance from the CEJ to the ABC was observed, using a stereomicroscope (Leica EZ4HD, Leica Microsystems AG) equipped with a digital camera. Recordings were made in the long axis of lingual root surfaces of the three molars. The photos were captured by SPOT RT software (Spot Diagnostic Instruments,).

### 2.5 Hematoxylin–eosin and TRAP staining

The mandibles were decalcified in a 10% EDTA solution for 4 weeks at 4°C, dehydrated with increasing concentrations of ethanol, and embedded in paraffin wax. Serial sagittal sections at a thickness of 5 μm were prepared and stained with hematoxylin–eosin (H&E) and tartrate‐resistant acid phosphatase (TRAP) staining. On the H&E‐stained sections, the micromorphometric alveolar bone loss was assessed by measuring the distance of CEJ‐ABC in the interdental regions (between the first and second molars). TRAP staining was performed via a leukocyte‐specific acid phosphatase kit (Sigma Aldrich). After TRAP staining, the sections (three sections per sample) were counterstained with hematoxylin. Active osteoclasts were defined as multinuclear TRAP‐positive cells in contact with the bone surface. The number of TRAP‐positive osteoclasts under the first and second molars on the surface of alveolar bone was counted. All the sections were analyzed under a light microscope (Nikon Eclipse 600).

### 2.6 Quantitative real‐time PCR

For the in vivo experiments, the left side of the mandibular bone was snap‐frozen in liquid nitrogen and kept at 80°C until use. The total RNA was extracted from the mandibular bone via an RNA Extraction Kit (TAKARA). The concentration and purity of extracted RNA were evaluated by a spectrophotometer (Thermo Scientific NanoDrop). After the cDNA was synthesized, real‐time quantitative PCR was performed using a SYBR^®^Premix Ex Taq^™^ II (TAKARA) on an ABI QuantStudio 3 PCR System (Applied Biosystems). The primer sequences for Runx2, ALP, OPG, RANKL, IL‐1β, IL‐6 and TNF‐ɑ, MnSOD, PGC‐1α and GAPDH are listed in Table 1. The CT values of detected genes were calculated in relation to GAPDH by the 2^−∆∆CT^ method.

**TABLE 1:** Primer sequences for real‐time qPCR analysis of the mRNA expression

Genes	Forward primer	Reverse primer
*Runx2*	AGCGGACGAGGCAAGAGTTT	AGGCGGGACACCTACTCTCATA
*ALP*	TCGGGACTGGTACTCGGATAAC	GTTCAGTGCGGTTCCAGACATAG
*OPG* (in vivo)	CCAGATGGGTTCTTCTCAGGTG	GTCCACCAAAACACTCAGCCAA
*RANKL*	CCATCGGGTTCCCATAAAGTCA	CAGTTTTTCGTGCTCCCTCCTT
*MnSOD*	TCCCAGACCTGCCTTACGACT	CCCTTAGGGCTCAGGTTTGTC
*PGC−1α*	TGTTCGCAGGCTCATTGTTG	GCTTGACTGGCGTCATTCGG
*IL−1β*	CACTACAGGCTCCGAGATGAAC	TCCATCTTCTTCTTTGGGTATTGC
*IL−6*	CCCCAATTTCCAATGCTCTCC	CGCACTAGGTTTGCCGAGTA
*TNF‐α*	ACCCTCACACTCACAAACCA	ATAGCAAATCGGCTGACGGT
*GAPDH*	CCTCGTCCCGTAGACAAAATG	TGAGGTCAATGAAGGGGTCGT
*SIRT3*	TGCTACTCATTCTTGGGACCT	GCTGGACCACATCTTTCCTT
*OPG* (in vitro)	ACCCAGAAACTGGTCATCAGC	CTGCAATACACACACTCATCACT
*Ndufs4*	CTGCCGTTTCCGTCTGTAGAG	TGTTATTGCGAGCAGGAACAAA
*Actin*	GGCTGTATTCCCCTCCATCG	CCAGTTGGTAACAATGCCATGT

For the in vitro experiments, the total RNA was extracted from shCtrl and shSIRT3 group cells using Trizol (Life Technologies), and cDNA samples containing 1000 ng RNA were synthesized using the iScript TM reverse Transcription Supermix (Bio‐rad). The PCR reaction was performed with iTaqTM Μniversal SYBR Green Supermix (Bio‐rad) and run in a CFX Connect quantitative PCR instrument. Gene expression results were expressed as arbitrary units relative to the expression of Actin. The primer sequences for SIRT3, OPG, Ndufs4, and Actin are listed in Table 1. The calculations were performed following the 2^−ΔΔCT^ method, where the Ctrl was calculated as the average CT value deriving from the Ctrl group.

### 2.7 MitoSOX staining

The mitochondrial ROS level in gingival tissues was measured by MitoSOX Red, a specific fluorochrome that selectively reacts with superoxide in the mitochondria. Fresh gingivae around the mandibular first molars were harvested and immediately incubated with 5 μM MitoSOX Red (Molecular Probes, Thermo) at 37°C for 30 min and protected from light. Then, the gingivae were prepared for the frozen section procedure. Meanwhile, the nuclei were stained with DAPI (Invitrogen). Fluorescent images were obtained using a Laser Scanning Confocal Microscope (LSCM, OLYMPUS FV3000) and quantified using the NIH Image J software. The intensity was normalized to the young wild‐type (WT) group.

### 2.8 NAD^+^/NADH Measurements

Gingival levels of NAD^+^ and NADH were measured using tissue homogenates with the EnzyChrom NAD^+^/NADH Assay Kit, according to the manufacturer's protocol (#E2ND‐100, Bioassay Systems). Optical density at 565 nm was recorded at time zero and at 15 min using a 96‐well plate reader spectrophotometer. The NAD^+^ and NADH concentrations were determined by comparing the difference in absorbance with standard curves, and they were normalized to the WT‐young group.

### 2.9 Immunohistochemistry analysis

Gingival tissues were fixed, immersed in paraffin and processed for sectioning. The sections were dewaxed and rehydrated. After antigen retrieval, all sections were treated with 3% hydrogen peroxidase for 30 min to block endogenous peroxidase activity. Then they were stained with primary antibodies against HO‐1, PGC‐1α, MnSOD, and CypD at 4°C overnight. Each primary antibody was used at a 1:200 dilution following the protocol. Subsequently, tissue sections were incubated with anti‐rabbit or anti‐mouse IgG antibodies for 30 min at room temperature. The detection of primary antibodies was visualized using a DAB‐horseradish peroxidase substrate system. Counterstaining was performed with hematoxylin. The immune reaction was visualized under a light microscope (Nikon Eclipse 600).

### 2.10 Immunofluorescence microscopy and mitochondrial morphology analysis

After treatment as described above, MC3T3‐E1 cells were seeded on glass coverslips in 6‐well plates and incubated overnight at 37°C. The next day, 2 ml of MitoTracker (Life Technologies) were added, followed by further incubation for 20–30 min at 37°C. After three washes with PBS, cells were fixed with 4% paraformaldehyde in PBS at room temperature for 20 min. Cells were next permeabilized with 2 ml IF washing solution for 1–2 min, washed twice with PBS, and incubated with IF blocking solution (2% BSA, 0.5% Saponin). Then the nuclei were stained with DAPI (Invitrogen). Coverslips were next washed with PBS and transferred from H2O onto a drop of ProLong Antifade Gold (Life Technologies). The quantitative analysis of mitochondrial morphology was performed using a morphometric application programmed on ImageJ^®^ (National Institutes of Health).^25^ This application determines the mean area, perimeter, major radius, and minor radius of cell mitochondria. Mitochondrial fusion/fission was evaluated using the form factor (FF = 4π × area/perimeter^2^) and the aspect ratio (AR = major radius/minor radius)^26^ after top hat filtering was applied to the raw images to remove noise and to obtain a precise definition of the mitochondrial morphology.

### 2.11 Measurement of the oxygen consumption rate

The oxygen consumption rate (OCR) of MC3T3‐E1 osteoblastic cells was measured with a Seahorse XFe24 analyzer (Agilent Technologies). The cells were seeded on a cell culture microplate for XFe24 Analyzer at a density of 20 000 cells/well the day before the experiments. On the test day, the medium was replaced with a commercially available Base Medium (Agilent) supplemented with 10 mM glucose, 1 mM pyruvate, and 2 mM glutamine. The OCR was measured following the manufacturer's instructions of the Seahorse XF Cell Mito Stress Test Kit (Agilent). The respiratory rates are reported as oxygen flux per 10 000 cells (pmol O_2_/min/cell^*^10^4^).

### 2.12 Statistics

Data are presented as mean ± SD. The statistical analysis was conducted with Student's *t*‐test for pairwise comparison and a one‐way ANOVA followed by Turkey's post hoc test for multiple comparisons. All analyses were performed using GraphPad Prism 7.0 software (Graphpad Software). Statistical significance was determined as *p* < .05.

## 3 RESULTS

### 3.1 Decreased SIRT3 protein abundance with age was related to hyperacetylation and a reduced NAD^+^/NADH ratio in alveolar bone

**FIGURE 1:**
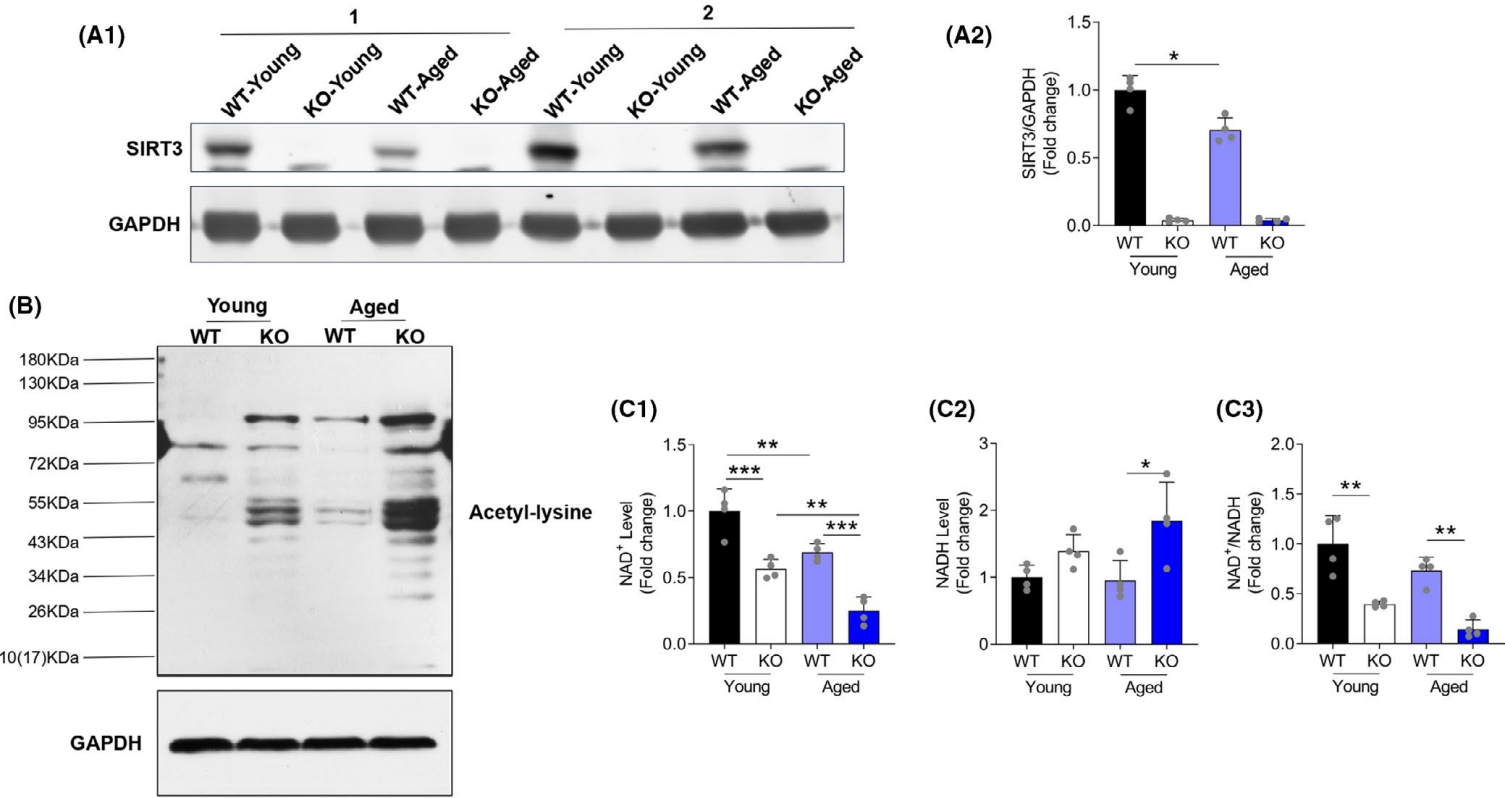
Sirtuin 3 expression decreased with age and protein hyperacetylation in SIRT3 KO mice. (A1, A2) Representative blots and quantification of protein expression of SIRT3. All data were normalized to GAPDH. (B) Acetylation level of the alveolar bone was examined by western blot. (C1‐C3) The NAD^+^, NADH, and NAD^+^/NADH ratio of gingival tissues were measured and normalized to the young WT group. Data are presented as mean ± SD for four mice in each group (*p < .05, **p < .01, ***p < .001 for indicated comparisons)

Sirtuin 3 is a major mitochondrial deacetylase that targets many enzymes involved in central metabolism.^1^ As age increased, we observed a decline of protein abundance of SIRT3 in alveolar bone (Figure 1A1‐A2), which was accompanied with an increased protein acetylation modification (Figure 1B), suggesting that SIRT3 may play a role in the pathogenesis of age‐related periodontal disease. SIRT3‐KO further enhanced the protein acetylation status of the alveolar bone after aged (Figure 1B). Since nicotinamide adenine dinucleotide (NAD^+^) is the cofactor of SIRT3 and serves as a key player in cellular metabolism, we sought to identify whether the reduced SIRT3 expression in maxillary bone was connected to dysregulated NAD^+^ biology. Indeed, the NAD^+^ level was reduced in the aged group, and SIRT3‐KO exacerbated the depletion of NAD^+^ in the alveolar bone at both ages (Figure 1C1). As compared to the young WT mice, the NADH level was slightly increased in the young SIRT3‐KO and attained a statistical difference in the aged SIRT3‐KO, resulting in a downregulation of the NAD^+^/NADH ratio in both young and aged SIRT3‐KO groups (Figure 1C2‐C3). Taken together, these data showed that SIRT3 deficiency resulting from age was related to hyperacetylation and a reduced NAD^+^/NADH ratio in alveolar bone.

### 3.2 Sirtuin 3 deficiency exacerbated age‐related periodontal bone resorption

**FIGURE 2:**
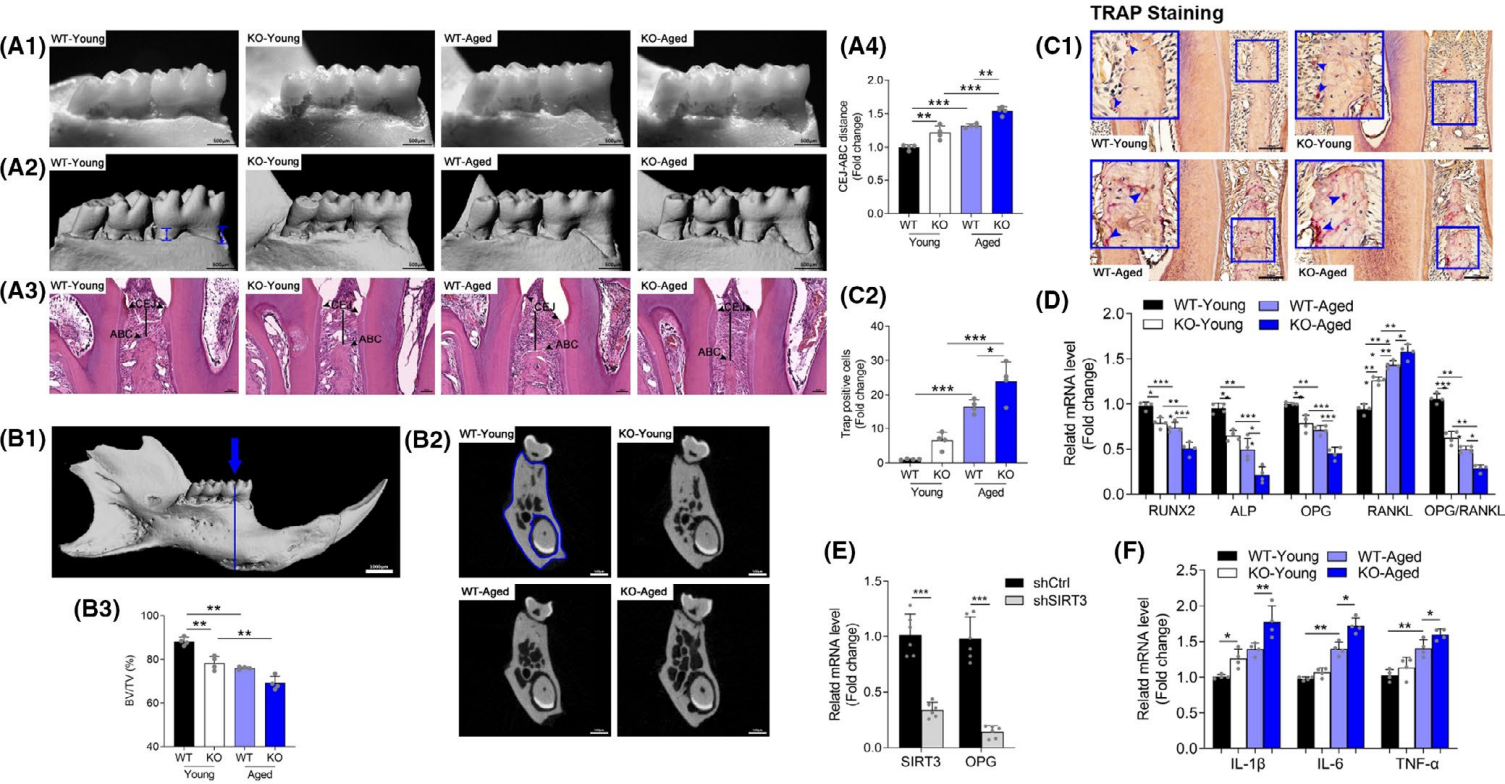
Sirtuin 3 deficiency aggravated age‐related alveolar bone loss. (A1‐A3) The distance between the cemento‐enamel junction (CEJ) and alveolar bone crest (ABC) represented alveolar bone loss, which was assessed in three ways: recordings kept via a stereomicroscope (Scale bar = 500 μm), three‐dimensional images (Scale bar = 500 μm), and H&E staining (Scale bar = 50 μm). Arrows in the images indicate the defined CEJ and ABC. (A4) The quantitative data of linear distances were obtained through μCT imaging. (B1) Lingual view of the mandible as seen on micro‐CT (Scale bar = 1000 μm). The morphological analysis centered on the furcation zone between the mesial and distal roots of the first mandibular molar (blue arrow and line). (B2) Area marked (section of bone, between the blue lines) represented the region of interest (ROI) evaluated for comparison between groups. Representative images of the cross‐sectional transverse scans in different groups (Scale bar = 500 μm). (B3) Bone volume per total volume (BV/TV, %) was quantified within the ROI. Tartrate‐resistant acid phosphatase (TRAP) staining to detect osteoclasts was performed on the tissue sections. (C1) Representative tissue sections from four groups were shown (Scale bar = 100 μm). (C2) Multinucleated TRAP‐positive osteoclasts were quantified. (D) Gene expression of Runx2, ALP, OPG, RANKL by RT‐qPCR analysis. The mRNA expression was normalized to GAPDH in each group. (E) Murine MC3T3‐E1 osteoblastic cells were incubated with control or SIRT3^−/−^ shRNA for 48 h. Then the gene expression level of SIRT3 and OPG were analyzed by RT‐qPCR. The mRNA expression was normalized to ACTIN in each group. (F) Gene expression of interleukin (IL)‐1β, IL‐6 and tumor necrosis factor‐ɑ by RT‐qPCR analysis. The mRNA expression was normalized to GAPDH in each group. Data are presented as mean ± SD for four mice in each group (*p < .05, **p < .01, ***p < .001 for indicated comparisons)

To understand the effect of reduced SIRT3 expression on alveolar bone loss, we measured alveolar bone architecture and morphology. The linear measurement of the three‐dimensional images indicated that the CEJ‐ABC distance was significantly increased in the aged WT mice in comparison with the young WT group (Figure 2A1‐A4). The SIRT3‐KO group exhibited high CEJ‐ABC distance at young age, which was further increased as they aged, suggesting that SIRT3 deficiency exacerbates age‐related periodontal bone loss. Similar findings were observed in the H&E staining images (Figure 2A3). Moreover, the alveolar bone architecture was determined by the micro‐CT evaluation of tomographic cross‐sectional slices taken within the furcation zone (Figure 2B1). The micro‐CT images of the mandibles showed morphological abnormalities in the aged WT and SIRT3‐KO mice, as compared to the young WT, including increases in bone marrow cavities and decreased trabecular connectivity (Figure 2B2). The calculated BV/TV of the trabecular bone was significantly lower under aged or SIRT3‐KO or both conditions, indicating that SIRT3 deficiency decreases trabecular connectivity (Figure 2B3).

Through TRAP staining, we found only a few osteoclasts in the Young WT group but many more in the aged and SIRT3‐KO groups. The highest osteoclast abundance was observed in the aged SIRT3‐KO mice, indicating that aging and SIRT3‐KO may synergistically contribute to osteoclastogenesis (Figure 2C1‐C2). In addition, we evaluated the expression of gene markers involved in osteogenic differentiation (Figure 2D). The mRNA levels of Runx2 and ALP were decreased in aged WT, and further reduced when combined with SIRT3‐KO. As regulators of osteoclastogenesis and bone inflammation, the gene expression of OPG was suppressed while the RANKL was elevated under aged or SIRT3‐KO or both conditions, resulting in decreased OPG/RANKL ratio. To confirm the impact of SIRT3 on osteoclastogenesis, we knocked down SIRT3 in vitro with SIRT3^−/−^ shRNA. Consistently, we found the mRNA level of OPG was significantly decreased in the shSIRT3 group (Figure 2E). In order to investigate whether the inflammatory response occurred within the alveolar bone tissues, we measured the gene expression level of three proinflammatory cytokines—interleukin‐1 (IL‐1), IL‐6, and tumor necrosis factor‐α (TNF‐α)—which have a central role in periodontal tissue destruction.^27^ Also, it is well known that IL‐1β stimulates bone loss and has an inhibitory effect to bone formation.^28^ We observed an approximately 20% increase in the IL‐1β expression in the young SIRT3‐KO group compared with the young WT, which was further increased in aged mice (Figure 2F). Both IL‐6 and TNF‐α mRNA levels showed a significant increase in the aged SIRT3‐KO group, compared to the young SIRT3‐KO group. Previous studies showed that those pro‐inflammatory cytokines elicit tissue destruction and bone resorption by stimulating collagenase and the RANKL, which induces osteoclast differentiation.^29^, ^30^ Together, these observations suggested that SIRT3 deficiency exacerbated age‐related periodontal bone resorption.

### 3.3 Sirtuin 3 deficiency induced oxidative damage in gingival tissues

**FIGURE 3:**
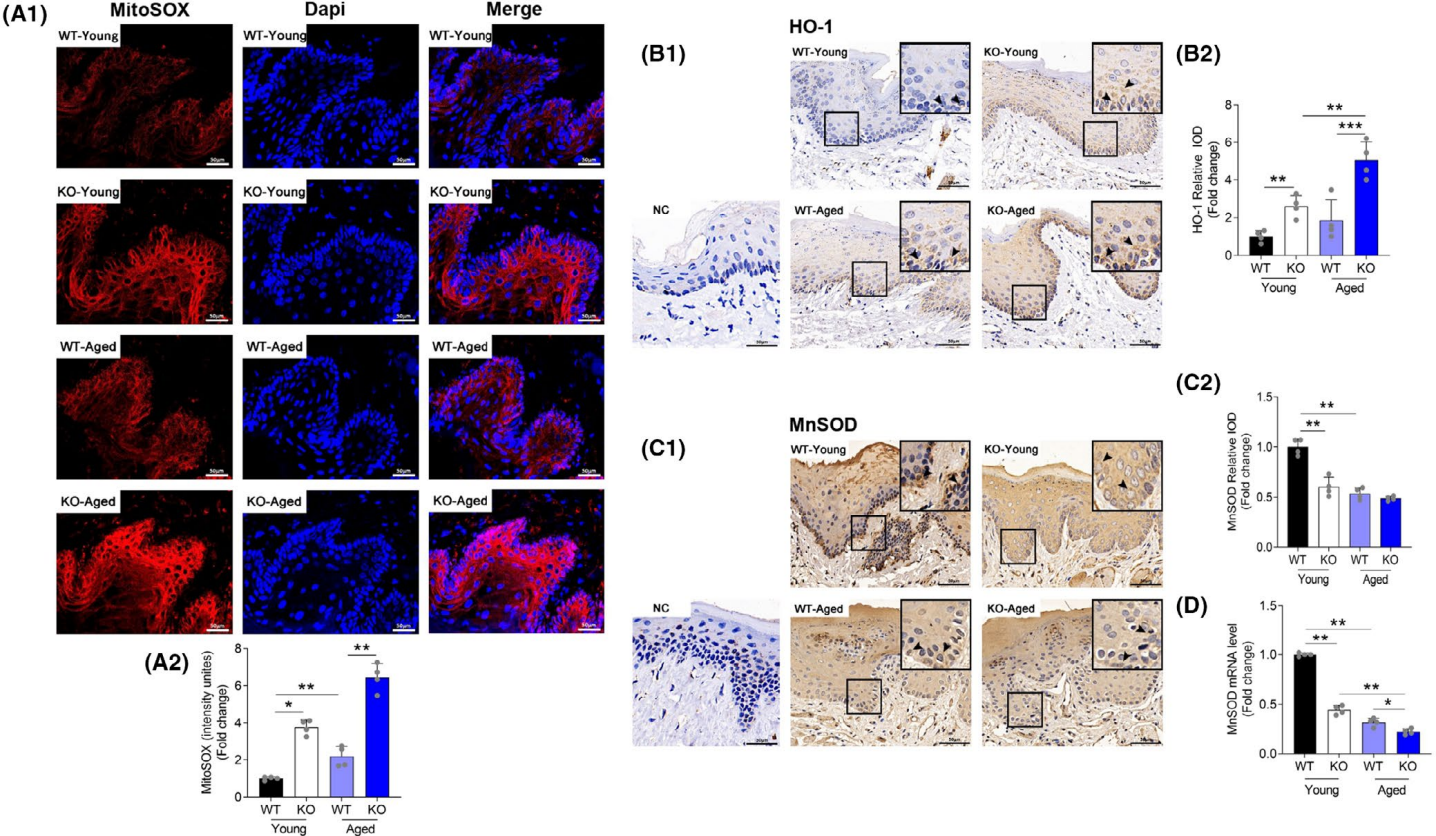
Loss of SIRT3‐induced oxidative damage in periodontal tissues. (A1) Representative images of MitoSOX red staining (Scale bar = 50 μm). The intensity was normalized to the young WT group. (A2) Data for the relative changes in MitoSOX Red fluorescence. (B1) Immunochemistry evaluation of HO‐1 of the gingival tissues (Scale bar = 50 μm, NC: negative control). The brown color indicates positive cells (arrows). (B2) Level of total ROS was assessed by HO‐1 staining intensity. (C1) Immunochemistry evaluation of MnSOD of the gingival sections (Scale bar = 50 μm, NC: negative control). The brown color indicates positive cells (arrows). (C2) Quantitative analysis of the protein expression in the periodontal area. (D) Gene expression of MnSOD by RT‐qPCR analysis. Data are presented as mean ± SD for four mice in each group (*p < .05, **p < .01, ***p < .001 for indicated comparisons)

To determine whether oxidative stress contributed to the pathogenesis in periodontal tissues due to SIRT3 deficiency, we measured the mitochondrial ROS in gingiva by MitoSOX staining and the total ROS level through HO‐1 IHC staining.^31^ A stronger red fluorescence was detected in the SIRT3‐KO relative to WT at both young and old age (Figure 3A1–A2). Of note, there was a 2.95‐fold increase of fluorescence intensity in the aged SIRT3‐KO when compared to the aged WT. Moreover, the intensity of HO‐1 staining was significantly enhanced in gingival epithelial spikes of the aged SIRT3‐KO, followed by the young SIRT3‐KO (Figure 3B1–B2). MnSOD, a downstream target gene of SIRT3, is an antioxidant enzyme that eliminate ROS in mitochondria.^32^ In our study, MnSOD expression was reduced in the aged and both SIRT3‐KO groups in comparison with the young WT group (Figure 3C1–C2). Consistent with the findings of IHC staining, the gene expression of MnSOD was also suppressed (Figure 3D). Taken together, these data suggested an overproduction of mitochondrial ROS in SIRT3‐deficient gingival tissues.

### 3.4 Sirtuin 3 deficiency influenced mitochondrial dynamics in periodontal tissues

**FIGURE 4:**
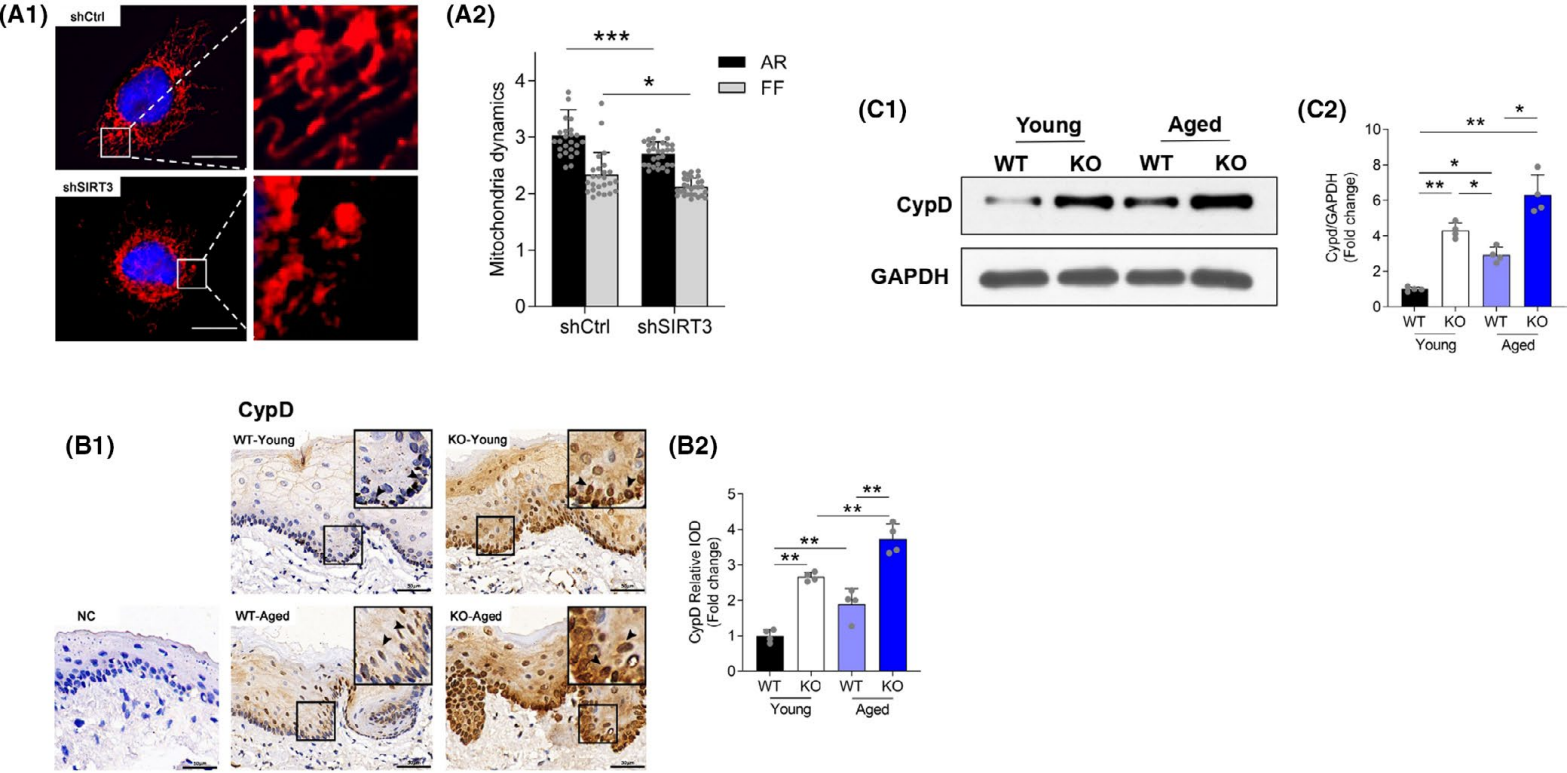
Sirtuin 3 deficiency promoted mitochondrial fission. (A1) Representative images of MitoTracker Red staining (Scale bar = 25 μm). (A2) Mitochondrial network architecture assessed using MitoTracker Red and calculation of aspect ratio (AR) and form factor (FF). (B1) Immunochemistry analysis of CypD in gingival tissues (Scale bar = 50 μm, NC: negative control). The brown color indicates positive cells (arrows). (B2) Assessment of the CypD staining intensity. (C1, C2) Representative blots and quantification of protein expression of CypD. Data are presented as mean ± SD for four mice in each group (*p < .05, **p < .01, ***p < .001 for indicated comparisons)

To test whether the increased mitochondrial ROS has measurable effects, we quantified mitochondrial morphology with SIRT3 knockdown MC3T3‐E1 osteoblastic cells. Mitochondrial fusion/fission was evaluated using the form factor (FF = 4π × area/perimeter^2^) and the aspect ratio (AR = major radius/minor radius). We found both the AR and FF significantly decreased in the SIRT3 knockdown cells, indicating that SIRT3 deficiency promotes mitochondrial fission (Figure 4A1–A2). Evidence suggests that SIRT3 regulates mitochondrial fission via deacetylation of CypD.^21^ Also, the CypD‐dependent mPTP opening is responsible for ROS‐induced cellular damage.^33^, ^34^ In this study, we used IHC staining and observed increased CypD protein expression in the gingival tissues of either young SIRT3‐KO or aged mice (Figure 4B1–B2). This finding was further confirmed by western blot and indicated that the protein abundance of CypD increased to 4.30‐fold in the young SIRT3‐KO group and 6.30‐fold in the aged SIRT3‐KO group, as compared to the young WT group (Figure 4C1–C2). Taken together, these results suggested that SIRT3 can influence the mitochondrial morphology and CypD expression level in periodontal tissues.

### 3.5 Sirtuin 3 deficiency suppressed mitochondrial biogenesis in periodontal tissues

**FIGURE 5:**
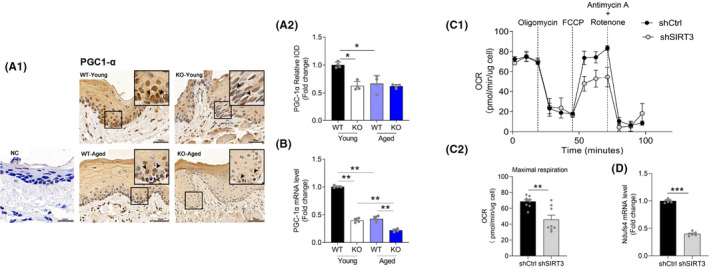
Sirtuin 3 deficiency suppressed mitochondrial biogenesis. (A1) Representative figures of PGC1‐α immunohistochemical staining in gingival tissues (Scale bar = 50 μm, NC: negative control). The brown color indicates positive cells (arrows). (A2) Quantitative analysis of PGC1‐α expression in the periodontal area. (B) Gene expression of PGC1‐α by RT‐qPCR analysis. (C1) Oxygen consumption rate (OCR) normalized to protein concentration. Following basal respiration, cells were treated sequentially with 1 μM Oligomycin, 2 μM FCCP, Antimycin A 1 + 1 μM Rotenone. (C2) Bar graphs of (C1) representing maximum respiration. (D) Gene expression of Ndufs4 by RT‐qPCR analysis. Data are presented as mean ± SD for four mice in each group (*p < .05, **p < .01, ***p < .001 for indicated comparisons)

To test whether SIRT3 deficiency influences mitochondria function, we chose to test PGC‐1α, which is considered to be a major regulator of mitochondrial biogenesis, and reduction in its levels has been related to mitochondrial dysfunction.^35^ The IHC staining data showed that the PGC‐1α protein expression was significantly decreased in the gingival tissues of the aged WT and both SIRT3‐KO groups as compared to the young WT (Figure 5A1–A2). Consistently, the mRNA level of PGC‐1α also decreased in aged and/or SIRT3‐KO mice (Figure 5B). To further investigate the functional role of SIRT3 in mitochondrial metabolism, we measured OCR through Seahorse analysis (Figure 5C1). SIRT3 knockdown MC3T3‐E1 osteoblastic cells exhibited a significant reduction in maximal OCR in comparison with the shCtrl group (Figure 5C2), indicating that SIRT3 deficiency impacts mitochondrial respiration and the oxidative phosphorylation (OXPHOS) system. The Ndufs4 gene encodes a nuclear‐encoded accessory subunit of the mitochondrial membrane respiratory chain NADH dehydrogenase (complex I, or NADH:ubiquinone oxidoreductase), which removes electrons from NADH and passes them to the electron acceptor ubiquinone.^36^, ^37^ We found that SIRT3 knockdown significantly down‐regulated the Ndufs4 mRNA level (Figure 5D), implying that SIRT3 may decrease the NAD^+^/NADH ratio by regulating complex I function. Taken together, these data suggested that SIRT3 deficiency reduced the PGC‐1α level and mitochondrial respiration, which may suppress mitochondrial biogenesis during aging in periodontal tissues.

## 4 DISCUSSION

**FIGURE 6:**
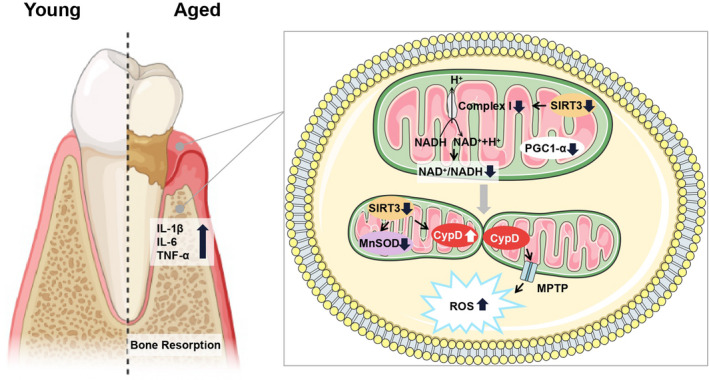
Schematic model of the results

The increased prevalence and severity of poor periodontal health have long been associated with advancing age. The present study assessed the functional role of SIRT3 in age‐related periodontal disease and the underlying mechanisms. We found a reduced SIRT3 expression and down‐regulated NAD^+^/NADH ratio in the periodontal tissues of aged mice. We also identified that SIRT3 is a potential player in regulating alveolar bone resorption and osteoclast formation. Deficiency in SIRT3 exacerbated age‐related periodontal bone loss (Figure 6).

Given that ROS are noxious byproducts of cellular metabolism mainly produced by the mitochondria, linking mitochondrial respiration with ROS effects is logical. ETC, especially complexes I and III, serves as the primary source and main target of ROS in mitochondria.^38^ The regulation of ROS by SIRT3 and ROS‐induced damage could be important mechanisms by which SIRT3 influences aging and the age‐related periodontal disease. MnSOD is the enzyme that converts harmful superoxide radicals to hydrogen peroxide in mitochondria.^39^, ^40^ SIRT3 directly deacetylates MnSOD in mitochondria, significantly enhancing its ability to scavenge ROS.^41^, ^42^

PGC1‐α is identified as an inducible upstream regulator of mitochondrial biogenesis that determines mitochondrial mass and function through its downstream targets NRF1 and NRF2.^11^ Moreover, PGC1‐α plays a vital role in controlling SIRT3 gene expression. SIRT3 may also upregulate PGC1‐α via a positive feedback mechanism.^43^ The structure of the mPTP includes the voltage‐dependent ion channel, adenine nucleotide translocator (ANT), and CypD. Binding of CypD to ANT initiates a tunnel‐like structure that connects the mitochondrial matrix with the cytosol.^44^ A previous study showed that SIRT3 suppressed excessive an mPTP opening and maintained mitochondrial membrane potential by activation of CypD via deacetylation.^45^ In this context, we found that the CypD expression was up‐regulated in aged mice or SIRT3‐KO mice, which emphasized the significance of SIRT3 in these processes.

The mechanisms for decreased SIRT3 expression during aging remain unclear. One possible reason could be the depletion of NAD^+^.^46^ Indeed, emerging evidence shows that the NAD^+^ level declines during aging, which decreased SIRT3 as a feedback, thereby leading to mitochondrial dysfunction and metabolic abnormalities.^47^, ^48^ In this study, we found SIRT3 knockdown may decrease complex I function, which could impact the NAD^+^/NADH ratio. A previous study provided direct evidence that oxidative stress‐induced physiological stem cell aging and tissue degeneration are rescued by upregulation of SIRT3.^49^ Furthermore, supplementation with NAD^+^ precursors like nicotinamide riboside, nicotinamide mononucleotide, and nicotinamide could ameliorate some aging‐related diseases.^50^, ^51^ Additionally, the mitochondrial NAD^+^ level and SIRT3 expression could also be increased by exercise, fasting, and caloric restriction.^52^, ^53^, ^54^

There are some limitations in our study. The aged mice in this study were 16 months old, which is beyond the range of middle‐aged mice (14–15 months) according to the definition from the Jackson Laboratory. However, they are still younger than mice defined as “old,” which ranges from 18 to 24 months of age. We did not perform rescue experiments to restore SIRT3 activity or expression to see whether that would ameliorate periodontal pathology. Also, most of our findings are based on observational data, more mechanistic studies are needed to uncover the underlying signaling pathways.

## 5 CONCLUSIONS

(1) Sirtuin 3 expression was decreased in the alveolar bone of the aged mice, accompanied by a reduced NAD^+^/NADH ratio and an elevated proteins acetylation level.

(2) Sirtuin 3‐KO exacerbated alveolar bone resorption and oxidative damage in gingival tissues.

(3) Sirtuin 3‐KO promoted mitochondrial fission and increased CypD expression in periodontal tissues.

(4) Sirtuin 3‐KO reduced PGC‐1α expression and mitochondrial respiration in periodontal tissues.

## CONFLICT OF INTEREST

None.

## Data Availability

The data that support the findings of this study are available from the corresponding author upon reasonable request.

## References

[1] Verdin E , Hirschey MD , Finley LW , Haigis MC . Sirtuin regulation of mitochondria: energy production, apoptosis, and signaling. Trends Biochem Sci. 2010;35:669‐675. 10.1016/j.tibs.2010.07.003 20863707 PMC2992946

[2] Newman HN . Diet, attrition, plaque and dental disease. Br Dent J. 1974;136:491‐497. 10.1038/sj.bdj.4803220 4531943

[3] Slots J . Periodontology: past, present, perspectives. Periodontology. 2013;2000(62):7‐19. 10.1111/prd.12011 23574461

[4] Harman D . The biologic clock: the mitochondria? J Am Geriatr Soc. 1972;20:145‐147. 10.1111/j.1532-5415.1972.tb00787.x 5016631

[5] Miquel J , Economos AC , Fleming J , Johnson JE Jr . Mitochondrial role in cell aging. Exp Gerontol. 1980;15:575‐591. 10.1016/0531-5565(80)90010-8 7009178

[6] Imai S , Armstrong CM , Kaeberlein M , Guarente L . Transcriptional silencing and longevity protein Sir2 is an NAD‐dependent histone deacetylase. Nature. 2000;403:795‐800. 10.1038/35001622 10693811

[7] Saunders LR , Verdin E . Sirtuins: critical regulators at the crossroads between cancer and aging. Oncogene. 2007;26:5489‐5504. 10.1038/sj.onc.1210616 17694089

[8] Schwer B , Verdin E . Conserved metabolic regulatory functions of sirtuins. Cell Metab. 2008;7:104‐112. 10.1016/j.cmet.2007.11.006 18249170

[9] He W , Newman JC , Wang MZ , Ho L , Verdin E . Mitochondrial sirtuins: regulators of protein acylation and metabolism. Trends Endocrinol Metab. 2012;23:467‐476. 10.1016/j.tem.2012.07.004 22902903

[10] Lombard DB , Alt FW , Cheng HL , et al. Mammalian Sir2 homolog SIRT3 regulates global mitochondrial lysine acetylation. Mol Cell Biol. 2007;27:8807‐8814. 10.1128/mcb.01636-07 17923681 PMC2169418

[11] Kincaid B , Bossy‐Wetzel E . Forever young: SIRT3 a shield against mitochondrial meltdown, aging, and neurodegeneration. Front Aging Neurosci. 2013;5:48. 10.3389/fnagi.2013.00048 24046746 PMC3764375

[12] Yu W , Dittenhafer‐Reed KE , Denu JM . SIRT3 protein deacetylates isocitrate dehydrogenase 2 (IDH2) and regulates mitochondrial redox status. J Biol Chem. 2012;287:14078‐14086. 10.1074/jbc.M112.355206 22416140 PMC3340192

[13] Ahn BH , Kim HS , Song S , et al. A role for the mitochondrial deacetylase Sirt3 in regulating energy homeostasis. Proc Natl Acad Sci U S A. 2008;105:14447‐14452. 10.1073/pnas.0803790105 18794531 PMC2567183

[14] Hirschey MD , Shimazu T , Goetzman E , et al. SIRT3 regulates mitochondrial fatty‐acid oxidation by reversible enzyme deacetylation. Nature. 2010;464:121‐125. 10.1038/nature08778 20203611 PMC2841477

[15] Shimazu T , Hirschey MD , Hua L , et al. SIRT3 deacetylates mitochondrial 3‐hydroxy‐3‐methylglutaryl CoA synthase 2 and regulates ketone body production. Cell Metab. 2010;12:654‐661. 10.1016/j.cmet.2010.11.003 21109197 PMC3310379

[16] Balaban RS , Nemoto S , Finkel T . Mitochondria, oxidants, and aging. Cell. 2005;120:483‐495. 10.1016/j.cell.2005.02.001 15734681

[17] Ito K , Hirao A , Arai F , et al. Reactive oxygen species act through p38 MAPK to limit the lifespan of hematopoietic stem cells. Nat Med. 2006;12:446‐451. 10.1038/nm1388 16565722

[18] Chapple IL . Reactive oxygen species and antioxidants in inflammatory diseases. J Clin Periodontol. 1997;24:287‐296. 10.1111/j.1600-051x.1997.tb00760.x 9178107

[19] Zorov DB , Juhaszova M , Sollott SJ . Mitochondrial reactive oxygen species (ROS) and ROS‐induced ROS release. Physiol Rev. 2014;94:909‐950. 10.1152/physrev.00026.2013 24987008 PMC4101632

[20] Kim SY , Shim MS , Kim KY , Weinreb RN , Wheeler LA , Ju WK . Inhibition of cyclophilin D by cyclosporin A promotes retinal ganglion cell survival by preventing mitochondrial alteration in ischemic injury. Cell Death Dis. 2014;5:e1105. 10.1038/cddis.2014.80 24603333 PMC3973219

[21] Hafner AV , Dai J , Gomes AP , et al. Regulation of the mPTP by SIRT3‐mediated deacetylation of CypD at lysine 166 suppresses age‐related cardiac hypertrophy. Aging. 2010;2:914‐923. 10.18632/aging.100252 21212461 PMC3034180

[22] Baines CP , Kaiser RA , Purcell NH , et al. Loss of cyclophilin D reveals a critical role for mitochondrial permeability transition in cell death. Nature. 2005;434:658‐662. 10.1038/nature03434 15800627

[23] Nakagawa T , Shimizu S , Watanabe T , et al. Cyclophilin D‐dependent mitochondrial permeability transition regulates some necrotic but not apoptotic cell death. Nature. 2005;434:652‐658. 10.1038/nature03317 15800626

[24] Tokunaga K , Seto H , Ohba H , et al. Topical and intermittent application of parathyroid hormone recovers alveolar bone loss in rat experimental periodontitis. J Periodontal Res. 2011;46:655‐662. 10.1111/j.1600-0765.2011.01386.x 21722135

[25] Theurey P , Tubbs E , Vial G , et al. Mitochondria‐associated endoplasmic reticulum membranes allow adaptation of mitochondrial metabolism to glucose availability in the liver. J Mol Cell Biol. 2016;8:129‐143. 10.1093/jmcb/mjw004 26892023

[26] Koopman WJ , Verkaart S , Visch HJ , et al. Inhibition of complex I of the electron transport chain causes O2‐. ‐mediated mitochondrial outgrowth. Am J Physiol Cell Physiol. 2005;288:C1440‐C1450. 10.1152/ajpcell.00607.2004 15647387

[27] Nikolopoulos GK , Dimou NL , Hamodrakas SJ , Bagos PG . Cytokine gene polymorphisms in periodontal disease: a meta‐analysis of 53 studies including 4178 cases and 4590 controls. J Clin Periodontol. 2008;35:754‐767. 10.1111/j.1600-051X.2008.01298.x 18673406

[28] Chung RM , Grbíc JT , Lamster IB . Interleukin‐8 and beta‐glucuronidase in gingival crevicular fluid. J Clin Periodontol. 1997;24:146‐152. 10.1111/j.1600-051x.1997.tb00483.x 9083897

[29] Seymour GJ , Gemmell E . Cytokines in periodontal disease: where to from here? Acta Odontol Scand. 2001;59:167‐173. 10.1080/000163501750266765 11501886

[30] Pan W , Wang Q , Chen Q . The cytokine network involved in the host immune response to periodontitis. Int J Oral Sci. 2019;11:30. 10.1038/s41368-019-0064-z 31685798 PMC6828663

[31] He Y , Zhang L , Zhu Z , Xiao A , Yu H , Gan X . Blockade of cyclophilin D rescues dexamethasone‐induced oxidative stress in gingival tissue. PLoS One. 2017;12:e0173270. 10.1371/journal.pone.0173270 28273124 PMC5342226

[32] Jiang DQ , Wang Y , Li MX , Ma YJ , Wang Y . SIRT3 in neural stem cells attenuates microglia activation‐induced oxidative stress injury through mitochondrial pathway. Front Cell Neurosci. 2017;11:7. 10.3389/fncel.2017.00007 28197079 PMC5281640

[33] Alam MR , Baetz D , Ovize M . Cyclophilin D and myocardial ischemia‐reperfusion injury: a fresh perspective. J Mol Cell Cardiol. 2015;78:80‐89. 10.1016/j.yjmcc.2014.09.026 25281838

[34] Feng D , Tang Y , Kwon H , et al. High‐fat diet‐induced adipocyte cell death occurs through a cyclophilin D intrinsic signaling pathway independent of adipose tissue inflammation. Diabetes. 2011;60:2134‐2143. 10.2337/db10-1411 21734017 PMC3142076

[35] Wang H , Bei Y , Lu Y , et al. Exercise prevents cardiac injury and improves mitochondrial biogenesis in advanced diabetic cardiomyopathy with PGC‐1alpha and Akt activation. Cell Physiol Biochem. 2015;35:2159‐2168. 10.1159/000374021 25896313

[36] van den Heuvel L , Ruitenbeek W , Smeets R , et al. Demonstration of a new pathogenic mutation in human complex I deficiency: a 5‐bp duplication in the nuclear gene encoding the 18‐kD (AQDQ) subunit. Am J Hum Genet. 1998;62:262‐268. 10.1086/301716 9463323 PMC1376892

[37] Emahazion T , Beskow A , Gyllensten U , Brookes AJ . Intron based radiation hybrid mapping of 15 complex I genes of the human electron transport chain. Cytogenet Cell Genet. 1998;82:115‐119. 10.1159/000015082 9763677

[38] Lustgarten MS , Bhattacharya A , Muller FL , et al. Complex I generated, mitochondrial matrix‐directed superoxide is released from the mitochondria through voltage dependent anion channels. Biochem Biophys Res Commun. 2012;422:515‐521. 10.1016/j.bbrc.2012.05.055 22613204 PMC3400138

[39] Spitz DR , Oberley LW . An assay for superoxide dismutase activity in mammalian tissue homogenates. Anal Biochem. 1989;179:8‐18. 10.1016/0003-2697(89)90192-9 2547324

[40] Zhang X , Ren X , Zhang Q , et al. PGC‐1alpha/ERRalpha‐Sirt3 pathway regulates DAergic neuronal death by directly deacetylating SOD2 and ATP synthase beta. Antioxid Redox Signal. 2016;24:312‐328. 10.1089/ars.2015.6403 26421366 PMC4761832

[41] Qiu X , Brown K , Hirschey MD , Verdin E , Chen D . Calorie restriction reduces oxidative stress by SIRT3‐mediated SOD2 activation. Cell Metab. 2010;12:662‐667. 10.1016/j.cmet.2010.11.015 21109198

[42] Tao R , Coleman MC , Pennington JD , et al. Sirt3‐mediated deacetylation of evolutionarily conserved lysine 122 regulates MnSOD activity in response to stress. Mol Cell. 2010;40:893‐904. 10.1016/j.molcel.2010.12.013 21172655 PMC3266626

[43] Padmaja Divya S , Pratheeshkumar P , Son YO , et al. Arsenic induces insulin resistance in mouse adipocytes and myotubes via oxidative stress‐regulated mitochondrial Sirt3‐FOXO3a signaling pathway. Toxicol Sci. 2015;146:290‐300. 10.1093/toxsci/kfv089 25979314

[44] Lemasters JJ , Theruvath TP , Zhong Z , Nieminen AL . Mitochondrial calcium and the permeability transition in cell death. Biochim Biophys Acta. 2009;1787:1395‐1401. 10.1016/j.bbabio.2009.06.009 19576166 PMC2730424

[45] Sun F , Si Y , Bao H , et al. Regulation of Sirtuin 3‐mediated deacetylation of cyclophilin D attenuated cognitive dysfunction induced by sepsis‐associated encephalopathy in mice. Cell Mol Neurobiol. 2017;37:1457‐1464. 10.1007/s10571-017-0476-2 28236057 PMC5630658

[46] Bochaton T , Crola‐Da‐Silva C , Pillot B , et al. Inhibition of myocardial reperfusion injury by ischemic postconditioning requires sirtuin 3‐mediated deacetylation of cyclophilin D. J Mol Cell Cardiol. 2015;84:61‐69. 10.1016/j.yjmcc.2015.03.017 25871830

[47] Gomes AP , Price NL , Ling AJ , et al. Declining NAD(+) induces a pseudohypoxic state disrupting nuclear‐mitochondrial communication during aging. Cell. 2013;155:1624‐1638. 10.1016/j.cell.2013.11.037 24360282 PMC4076149

[48] Scheibye‐Knudsen M , Mitchell SJ , Fang EF , et al. A high‐fat diet and NAD(+) activate Sirt1 to rescue premature aging in cockayne syndrome. Cell Metab. 2014;20:840‐855. 10.1016/j.cmet.2014.10.005 25440059 PMC4261735

[49] Brown K , Xie S , Qiu X , et al. SIRT3 reverses aging‐associated degeneration. Cell Rep. 2013;3:319‐327. 10.1016/j.celrep.2013.01.005 23375372 PMC3582834

[50] Long AN , Owens K , Schlappal AE , Kristian T , Fishman PS , Schuh RA . Effect of nicotinamide mononucleotide on brain mitochondrial respiratory deficits in an Alzheimer's disease‐relevant murine model. BMC Neurol. 2015;15:19. 10.1186/s12883-015-0272-x 25884176 PMC4358858

[51] Liu D , Pitta M , Jiang H , et al. Nicotinamide forestalls pathology and cognitive decline in Alzheimer mice: evidence for improved neuronal bioenergetics and autophagy procession. Neurobiol Aging. 2013;34:1564‐1580. 10.1016/j.neurobiolaging.2012.11.020 23273573 PMC3596471

[52] Shi T , Wang F , Stieren E , Tong Q . SIRT3, a mitochondrial sirtuin deacetylase, regulates mitochondrial function and thermogenesis in brown adipocytes. J Biol Chem. 2005;280:13560‐13567. 10.1074/jbc.M414670200 15653680

[53] Palacios OM , Carmona JJ , Michan S , et al. Diet and exercise signals regulate SIRT3 and activate AMPK and PGC‐1alpha in skeletal muscle. Aging. 2009;1:771‐783. 10.18632/aging.100075 20157566 PMC2815736

[54] Deng Y , Xie M , Li Q , et al. Targeting Mitochondria‐Inflammation Circuit by β‐Hydroxybutyrate Mitigates HFpEF. Circulation Research. 2021;128(2):232‐245. 10.1161/circresaha.120.317933 33176578

